# Recurrent Metastatic Colorectal Adenocarcinoma to the Thyroid Gland Presenting With Vocal Cord Paralysis and Inspiratory Stridor

**DOI:** 10.7759/cureus.42087

**Published:** 2023-07-18

**Authors:** David P Le

**Affiliations:** 1 Internal Medicine, University of South Alabama, Mobile, USA

**Keywords:** metastases to the thyroid, metastatic colorectal cancer, malignant thyroid nodule, elevated carcinoembryonic antigen (cea), immunohistochemical markers

## Abstract

The most common sites for metastases of colorectal cancer include the liver, lungs, brain, and regional lymph nodes. However, a limited number of reported cases describe colon cancer metastasis to the thyroid gland. Metastatic colorectal adenocarcinoma to the thyroid gland is rare. The majority of these cases with colon cancer metastases to the thyroid gland are diagnosed years after initial treatment of colon cancer. The discovery is usually made after routine surveillance imaging, and often patients have minimal or absent symptoms. We report a case of a recurrence of metastatic colorectal adenocarcinoma to the thyroid gland presenting with vocal cord paralysis and inspiratory stridor.

## Introduction

Around 1% of all thyroid neoplasms are considered to be of metastatic origin [1]. Lung, breast, and renal carcinomas are the common sources of metastases to the thyroid [2]. In a patient with a history of metastatic cancer, the recurrence of cancer to the thyroid gland remains a possibility. Thyroid nodules with malignant characteristics are diagnosed with fine needle aspiration (FNA) with histopathological analysis for diagnosis. With specific immunohistochemistry staining and techniques, clinicians can readily diagnose a primary recurrence of colonic cancer that may have spread to the thyroid gland.

Head and neck malignancy, primary thyroid cancers, and iatrogenic injury to the recurrent laryngeal nerve during surgeries are the most common causes of vocal cord paresis. However, metastatic colorectal cancer (CRC) to the thyroid is one rare cause that should be considered. Hoarseness, voice changes, and an enlarging throat mass may be presenting symptoms of a thyroid malignancy. The symptoms that may arise from a thyroid nodule can be derived from the anatomic relationship between the trachea, thyroid gland, and recurrent laryngeal nerve. The thyroid gland lies anterolateral to the trachea, and an enlarging mass may compromise airway. The recurrent laryngeal nerve lies posteromedially to the trachea and, when injured, can produce changes in the patient’s voice.

## Case presentation

**Figure 1:**
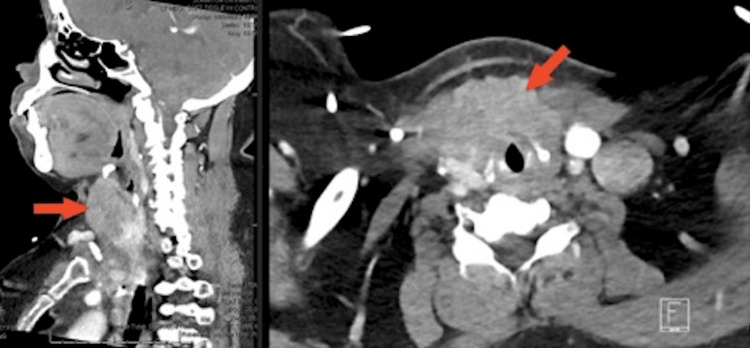
CT of the chest with contrast showing a heterogeneous thyroid lobe mass measuring 4.9 x 3.0 cm (arrows) without mass effect on the respiratory tract. CT, computed tomography

A 61-year-old female with a past medical history of invasive sigmoid colon adenocarcinoma with right upper lung lobe and peritracheal lymph node metastasis presented with a two-month history of worsening dyspnea, hoarseness, and throat pain. The patient’s medical history included a previous sigmoidectomy, nodal dissection, and chemoradiation of her stage 3 (pT2, pN1, M1) moderately differentiated colonic adenocarcinoma. She underwent surveillance colonoscopy and imaging 3 years after her initial diagnosis and management of her colon cancer with no recurrence of disease. She had no history of tobacco or alcohol use. The patient did not have a history of cancer in her family. She denied abdominal pain, weight gain/loss, heat/cold intolerance, abnormal bowel habit changes, or rectal bleeding. On lung examination, the patient had increased respiratory effort, and marked inspiratory stridor was noted. On physical examination, the patient had a firm neck mass extending to the suprasternal notch. The mass moved with swallowing, and there was no cervical or supraclavicular lymphadenopathy. Laboratory findings did not show anemia or abnormalities in the renal or liver function. However, carcinoembryonic antigen (CEA) was elevated to 57.7 ng/mL (normal range: 1.0-4.1 ng/mL). Thyroid function levels were normal. CT (computed tomography) imaging revealed an enlarging thyroid mass in front of the trachea and larynx. The thyroid mass was adjacent to the respiratory tract, and multiple bilateral pulmonary nodules were on CT concerning for disease progression (Figure 1).

**Figure 2:**
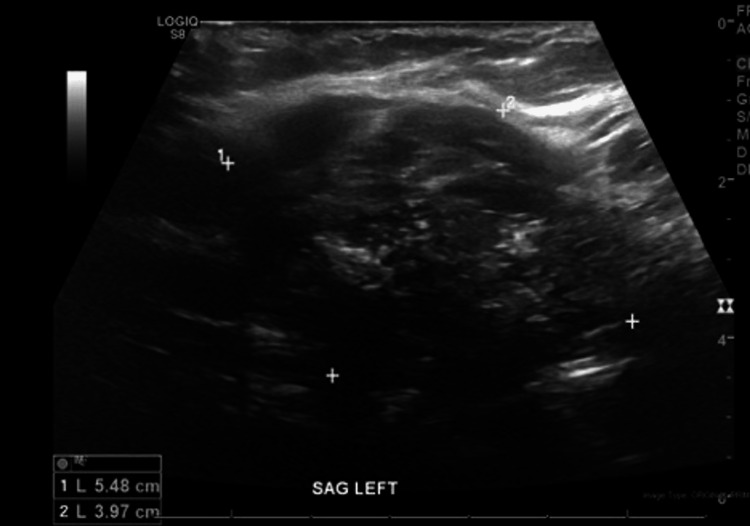
Thyroid ultrasound (sagittal view) of the left thyroid lobe with a solid, hypoechoic, irregular, heterogenous nodule with punctate echogenic foci, measuring 5.5 x 4.0 cm with extrathyroidal extension, suspicious for malignancy.

On ultrasound, the left thyroid nodule showed irregular features with extrathyroidal extension concerning for malignancy (Figure 2).

**Figure 3:**
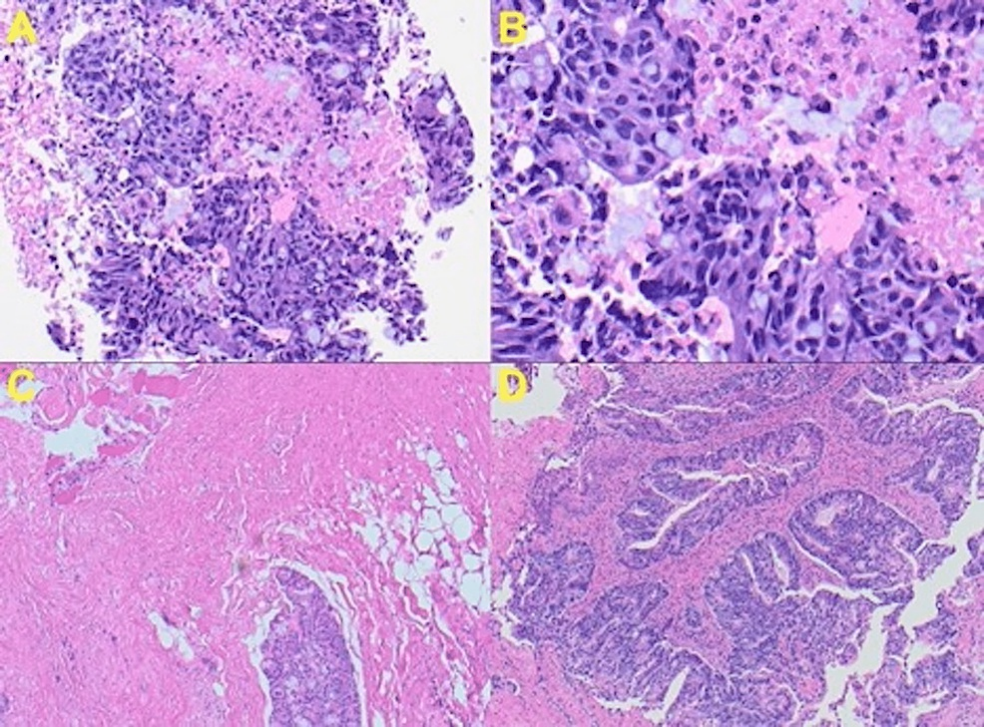
H&E stains of FNA aspiration cytology of the thyroid nodule: (A) Colonic adenocarcinoma with glands formation. Prominent nuclear atypia with mitotic figures. Sparse islands of thyroid tissue present (20x). (B) Mucin-producing carcinoma cells, thyroid colloids (40x). (C) Visible thyroid round follicles of colloid. Desmoplasia of colonic adenocarcinoma with adipose tissue (10x). (D) Infiltrative gland-forming colorectal adenocarcinoma, purple cytoplasm, rounded glands (20x). FNA, fine needle aspiration; H&E, hematoxylin and eosin

A fine needle aspiration cytology (FNAC) of the thyroid nodule was performed, which showed loose groups of columnar cells with mucin-producing glandular tissue visualized with sparse thyroid colloids (Figure 3). Atypical cells exhibited marked pleomorphism, and prominent and hyperchromatic nuclei with necrotic debris (Figure 3). Cells also displayed increased nuclear-to-cytoplasmic ratio, with increased irregular contours and cytoplasmic mucin-like contents.

**Figure 4:**
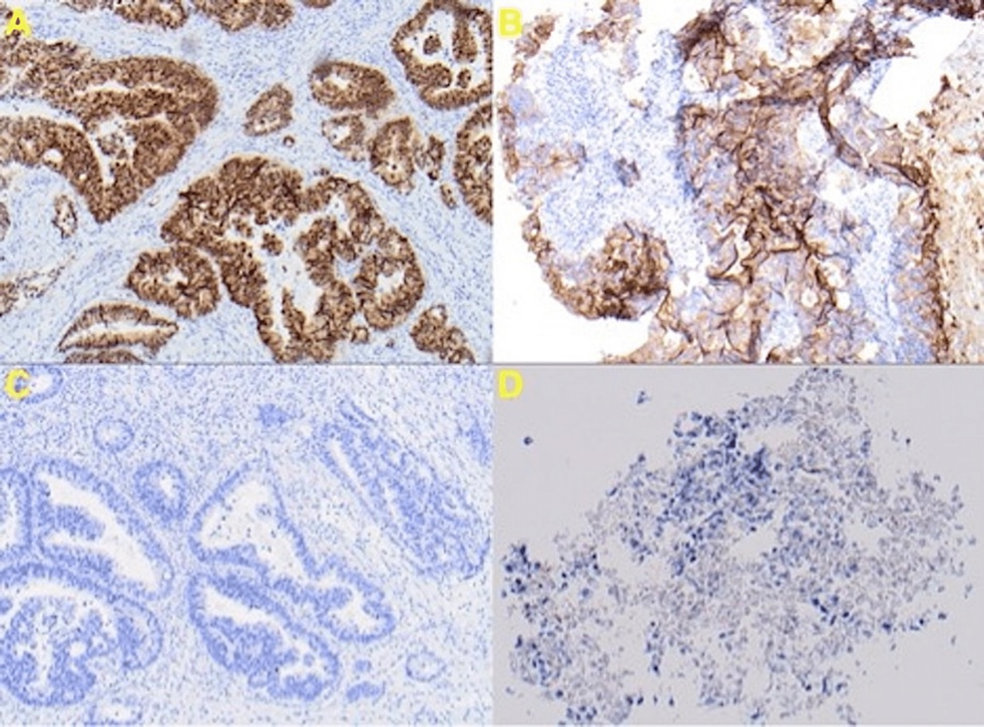
Immunoperoxidase stains of thyroid tissue. (A) Positive CDX-2 stain (20x). (B) Positive CK20 stain (10x). (C) CK7 negative stain (10x). (D) TTF-1 negative stain (20x). CDX-2, caudal-type homeobox 2; CK20, cytokeratin 20; CK7, cytokeratin 7; TTF-1, thyroid transcription factor 1

As shown in Figure 4, the immunohistochemistry study showed the tumor to be positive for cytokeratin 20 (CK20) and caudal-type homeobox 2 (CDX-2), and negative for cytokeratin 7 (CK7) and thyroid transcription factor 1 (TTF-1). This specific immunophenotype pattern is characteristic of colorectal carcinomas. Given the clinical and histopathological cytology results, a diagnosis of thyroid metastasis from primary colonic adenocarcinoma was made.

During the hospitalization, the patient’s worsening dyspnea and increased oxygen requirements prompted a consultation with a head and neck surgeon. Fiberoptic laryngoscopy was performed and showed complete airway obstruction visualized at the level of the vocal cords by the tumor mass. Both vocal cords were swollen, paralyzed, and fixed midline causing severe upper airway obstruction. The trachea was significantly narrow, and the patient underwent emergent tracheostomy. Biopsy of thyroid mass reconfirmed the FNA findings of adenocarcinoma of colorectal origin. Surgical resection was difficult due to the tumor involvement of adjacent structures including the cricoid and thyroid cartilage, with deep invasion of the cricothyroid muscle. With the recurrence of CRC metastasized to the thyroid, an aggressive growth pattern was suspected and a palliative treatment approach was planned. The patient received palliative radiation therapy to the neck and was subsequently started on systemic chemotherapy.

## Discussion

The incidence of metastases to the thyroid gland is estimated to be less than 3% [3]. Typical malignant tumors that tend to metastasize to the thyroid gland include the breast, lung, and renal carcinomas [4]. Colorectal carcinomas metastasizing to the thyroid gland is extremely rare. Metastases to the thyroid gland often present asymptomatically and are only incidentally discovered on follow-up scans for a patient’s primary tumor [5]. According to Coelho et al., metastases to the thyroid, if symptomatic, will commonly present with swelling and cervical mass, or have goiter-related symptoms [6]. Laboratory abnormalities such as thyroid dysfunction and an increase in tumor marker (CEA) are the first diagnostic clues of a metastatic carcinoma to the thyroid. Ultrasound, CT, and positron emission tomography (PET) imaging can be used to detect an abnormal thyroid nodule; however, only FNAC has high positive and negative predictive values of distinguishing between a primary thyroid lesion and a metastasis [6]. Iguchi et al. proposed that thyroid metastasis from a CRC can be detected earlier with the use of F-18 fluorodeoxyglucose (FDG) PET during routine search for distant metastases or local recurrence if CEA levels were elevated [7]. In addition, F-18 FDG PET has been useful to detect other metastatic sites of colon cancer in the liver, lungs, and lymph nodes [7]. A patient with secondary malignancy to the thyroid gland may mimic a primary anaplastic thyroid cancer with similar symptomatology of dyspnea and dysphonia [8]. Regardless of the latency period after a patient’s treated primary lesion, it is imperative to consider the possibility of tumor metastases to the thyroid especially in a patient with a history of metastatic cancer with a new thyroid mass, changes in voice, hoarseness, or stridor.

The thyroid gland is one of the most richly arterialized organs in the body, but it is surprising that metastases to the thyroid are exceedingly rare due to a variety of mechanisms. Metastatic tumor to the thyroid can either occur through direct extension from nearby tissue or through lymphovascular spread [9]. One hypothesis suggests that the high oxygen and iodine content in the thyroid gland prevents growth of tumor cells from other organs [10]. Another theory proposed that the rich vascularity of the thyroid gland prevents tumor cells from staying fixed in the thyroid gland [10].

Thyroid metastases can present months to years after initial diagnosis and treatment of the primary cancer [9]. A new thyroid nodule in the setting of a patient with previous malignant disease should prompt a clinician to perform further imaging and biopsy to confirm a newly diagnosed metastatic disease [6]. FNA with cytopathological evaluation can reliably diagnose both primary and metastatic thyroid neoplasms.

FNAC of the thyroid has been shown to be a reliable diagnostic tool to diagnose thyroid neoplasms [11]. Adenocarcinoma cells with a necrotic background reflect the primary tumor features of a well-differentiated colonic adenocarcinoma with tubular formation. Poorly differentiated tumors can be subtyped utilizing anti-cytokeratin and other antibodies. Immunohistochemical staining of markers for renal cell, lung, and thyroid carcinomas could be used to distinguish primary thyroid and metastatic neoplasms of the thyroid [12]. CK7, CK20, and TTF-1 are epithelial markers of lung and thyroid tissues [12]. Most colorectal adenocarcinomas will be positive for CK20, but negative for TTF-1 and CK7. CK7 is present in most lung adenocarcinomas, but rare in CRC [13]. Pathologists use the combination of CK7 and CK20 to differentiate between CRC and a primary lung cancer [13]. The majority of cases of colorectal adenocarcinomas will have negativity for CK7 and positivity for CK20 [14]. In comparison, according to Bejarano et al., thyroid neoplasms will be positive for CK7 but negative for CK20 in the majority of cases [14]. TTF-1 is another immunohistochemical marker that is highly sensitive and specific for pulmonary neoplasms. Dettmer et al. stated that CRC rarely stains for TTF-1 positivity. However, TTF-1 has a higher rate of immunoreactivity and has several advantages over traditional thyroglobulin immunostaining for poorly differentiated carcinomas and metastatic lesions [14]. CDX-2 is a marker for benign colonic epithelium and intestinal epithelium cells, and is expressed as well in most colorectal adenocarcinomas [15]. By utilizing a panel of immunohistochemical staining (+CDX-2, +CK20, -CK7, -TTF-1) in our case, we were able to correctly diagnose a metastatic colorectal adenocarcinoma to the thyroid gland.

Thyroid metastases are managed on an individual basis depending on the performative function of the patient, stage, and extent of the disease. Previous studies have shown that surgical management of thyroid gland metastases such as lobectomy, isthmectomy, and total thyroidectomy are viable options [9]. With widespread disease or severe complications, systemic chemotherapy or radiotherapy has been utilized in prior cases of thyroidal metastases. However, combination chemotherapy has questionable benefits due to poor penetration of chemotherapeutic drugs to the thyroid gland [6]. Patients with widespread metastases have extremely poor prognosis because of the difficulty of surgically resecting all tumor lesions, and it is unclear if surgical treatment will prolong a patient’s overall survival [9]. In a patient who has undergone a sigmoidectomy with subsequent development of metastasis to the thyroid gland, extensive spread with multiple extrathyroidal metastases will need to be managed with a palliative approach. Previous reports have shown that curative rates of thyroid metastases from a colorectal carcinoma are low and that patients do not do well even after commencement of chemotherapy [16]. Radiation and chemotherapy with palliative intent is usually reserved for patients with widespread disease. Localized radiotherapy, as in our case, is used in cases of life-threatening symptoms of dyspnea and dysphagia. Thyroidectomy is another option to preserve a patient’s quality of life functions of swallowing, respiration, and voicing [3].

## Conclusions

Metastatic colorectal adenocarcinoma to the thyroid gland presenting with vocal cord paralysis and inspiratory stridor is a rare occurrence. This case highlights the importance of having a low threshold of clinical suspicion of considering metastatic disease in the differential diagnosis of thyroid masses, particularly in patients with a history of metastatic colorectal adenocarcinoma. Severe wheezing, dyspnea, and hoarseness with concerns for vocal cord paralysis and inspiratory stridor should prompt urgent surgical evaluation and management. In our case, emergent tracheostomy and laryngoscopy were necessary for airway management and to avoid further respiratory compromise. Metastases to the thyroid gland are an increasing phenomenon that should be recognized by clinicians due to their aggressive growth pattern. Prompt FNA and biopsy of a new thyroid mass with accurate histopathological analysis can diagnose a primary CRC that has metastasized to the thyroid gland. Despite prior management of the patient's primary colorectal carcinoma and the prolonged interval from the time of her last treatment, her disease eventually recurred, emphasizing the poor prognosis associated with metastatic colorectal adenocarcinoma to the thyroid gland.
